# Descending necrotizing mediastinitis after pharyngeal fish bone impaction: a case report

**DOI:** 10.1093/omcr/omag177

**Published:** 2026-09-25

**Authors:** ES-Sddik El Bakri, Maryeme Lahjaouj, Maryeme Loudghiri, Walid Bijou, Yassine Oukessou, Sami Rouadi, Redallah Abada, Mohamed Roubal, Mohamed Mahtar

**Affiliations:** Department of Otorhinolaryngology (ENT), 20 August Hospital, Ibn Rochd University Hospital, 4 Rue Lahcen El Arjoune, Quartier des Hôpitaux, 20360 Casablanca, Morocco; Department of Otorhinolaryngology (ENT), 20 August Hospital, Ibn Rochd University Hospital, 4 Rue Lahcen El Arjoune, Quartier des Hôpitaux, 20360 Casablanca, Morocco; Department of Otorhinolaryngology (ENT), 20 August Hospital, Ibn Rochd University Hospital, 4 Rue Lahcen El Arjoune, Quartier des Hôpitaux, 20360 Casablanca, Morocco; Department of Otorhinolaryngology (ENT), 20 August Hospital, Ibn Rochd University Hospital, 4 Rue Lahcen El Arjoune, Quartier des Hôpitaux, 20360 Casablanca, Morocco; Department of Otorhinolaryngology (ENT), 20 August Hospital, Ibn Rochd University Hospital, 4 Rue Lahcen El Arjoune, Quartier des Hôpitaux, 20360 Casablanca, Morocco; Department of Otorhinolaryngology (ENT), 20 August Hospital, Ibn Rochd University Hospital, 4 Rue Lahcen El Arjoune, Quartier des Hôpitaux, 20360 Casablanca, Morocco; Department of Otorhinolaryngology (ENT), 20 August Hospital, Ibn Rochd University Hospital, 4 Rue Lahcen El Arjoune, Quartier des Hôpitaux, 20360 Casablanca, Morocco; Department of Otorhinolaryngology (ENT), 20 August Hospital, Ibn Rochd University Hospital, 4 Rue Lahcen El Arjoune, Quartier des Hôpitaux, 20360 Casablanca, Morocco; Department of Otorhinolaryngology (ENT), 20 August Hospital, Ibn Rochd University Hospital, 4 Rue Lahcen El Arjoune, Quartier des Hôpitaux, 20360 Casablanca, Morocco

**Keywords:** descending necrotizing mediastinitis, pharyngeal foreign body, cervical cellulitis, surgical drainage, type 2 diabetes mellitus

## Abstract

Descending necrotizing mediastinitis (DNM) is an uncommon but severe complication of deep neck infection. Most cases arise from odontogenic sources, whereas pharyngeal fish bone impaction is rare. We report the case of a 54-year-old man with poorly controlled type 2 diabetes mellitus who developed DNM 10 days after endoscopic removal of a fish bone lodged in the right vallecula. Flexible nasofibroscopy showed a residual vallecular mucosal lesion with marked supraglottic oedema, and contrast-enhanced cervicothoracic CT demonstrated extensive cervical infection with mediastinal spread. The patient underwent emergency cervicotomy with cervical and mediastinal drainage through a cervical approach, followed by broad-spectrum antibiotics, tracheostomy, and intensive care. He improved gradually with multidisciplinary care. This case underscores the need for early recognition, urgent surgical drainage, and coordinated management, especially in patients with poorly controlled diabetes, even after apparently successful foreign body removal.

## Introduction

Descending necrotizing mediastinitis (DNM) is a rare but life-threatening complication of deep neck infections, characterized by rapid extension of infection from cervical spaces into the mediastinum through fascial planes. Despite advances in imaging and critical care, mortality remains high, ranging from 9% to 40% depending on early recognition and extent of disease [1–3].

Foreign body ingestion, particularly fish bones, is a frequent ENT emergency. While most cases are benign, delayed diagnosis or mucosal injury may result in deep cervical infection and, rarely, mediastinal extension particularly in patients with poorly controlled diabetes mellitus [4, 5].

We report a case of cervicofacial cellulitis complicated by DNM following pharyngeal fish bone ingestion in context of poorly controlled diabetes mellitus, emphasizing diagnostic challenges and therapeutic management.

## Case presentation

A 54-year-old man with poorly controlled type 2 diabetes mellitus (fasting blood glucose, 3.8 g/L; HbA1c, 12%) presented to the emergency department with a 6-day history of odynophagia, hypersalivation, moderate dyspnoea, retrosternal pain, fever (39°C), and asthenia. Ten days before admission, he had undergone endoscopic removal of a fish bone impacted in the right vallecula. He reported no recent dental treatment, and oral examination revealed satisfactory dentition without evidence of dental caries or periodontal disease.

**Figure 1:**
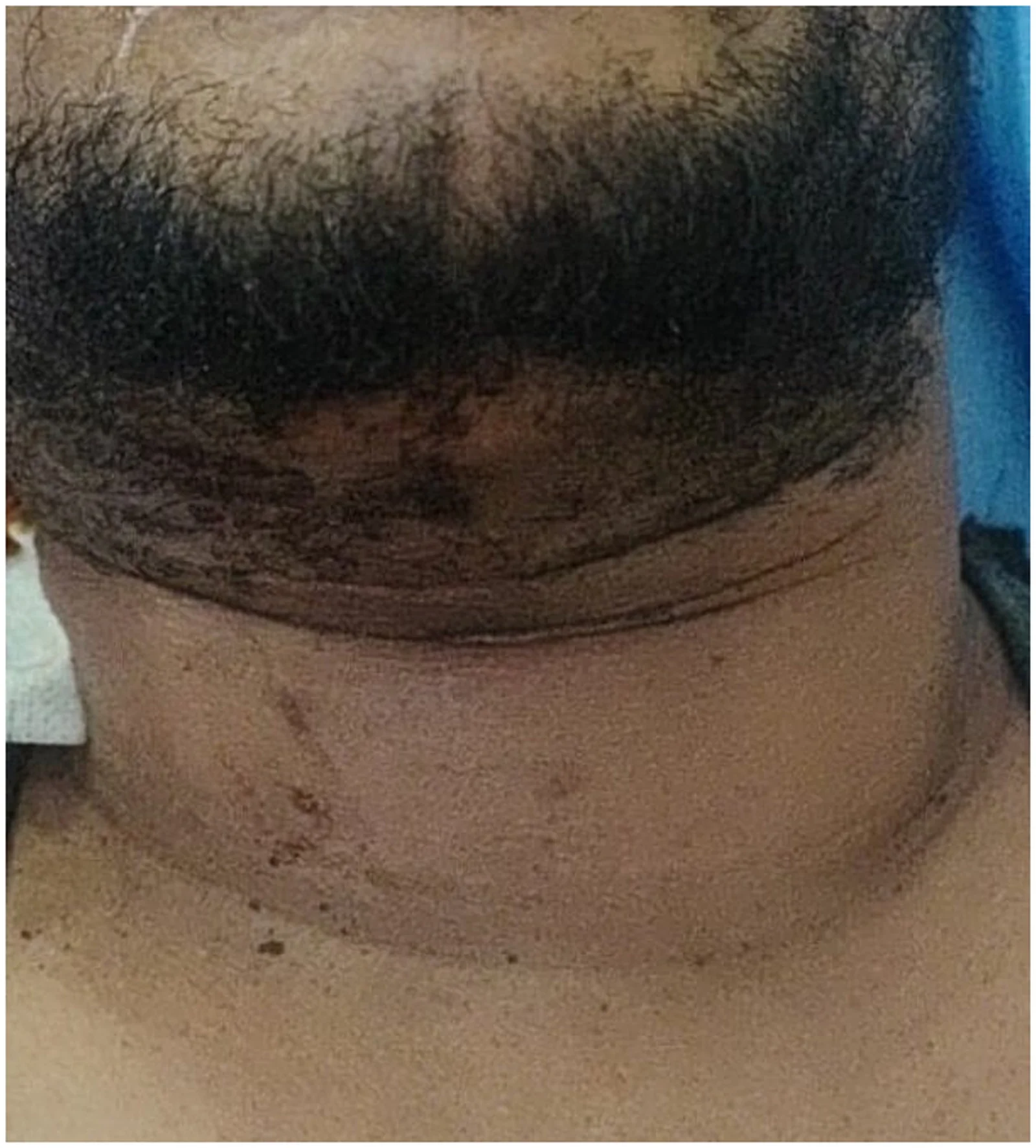
Preoperative photo of the neck demonstrating a diffuse anterior cervical swelling with inflammatory features.

On examination, he was febrile with diffuse anterior cervical swelling extending from the submental region to the supraclavicular fossae, associated with induration and subcutaneous crepitus (Fig. 1).

**Figure 2:**
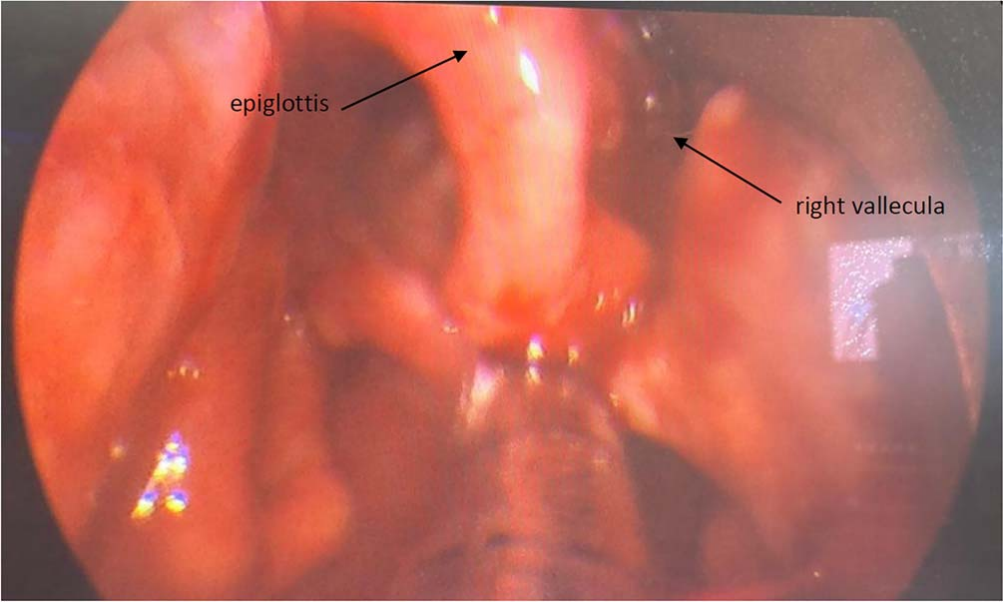
Nasofibroscopy demonstrated inflammatory changes of the supraglottic region, characterized by an oedematous and thickened epiglottis, along with a mucosal lesion involving the right vallecula*.*

Flexible nasofibroscopy revealed inflammatory changes in the right vallecula with diffuse oedema of the upper aerodigestive tract and mild airway narrowing (Fig. 2).

**Figure 3:**
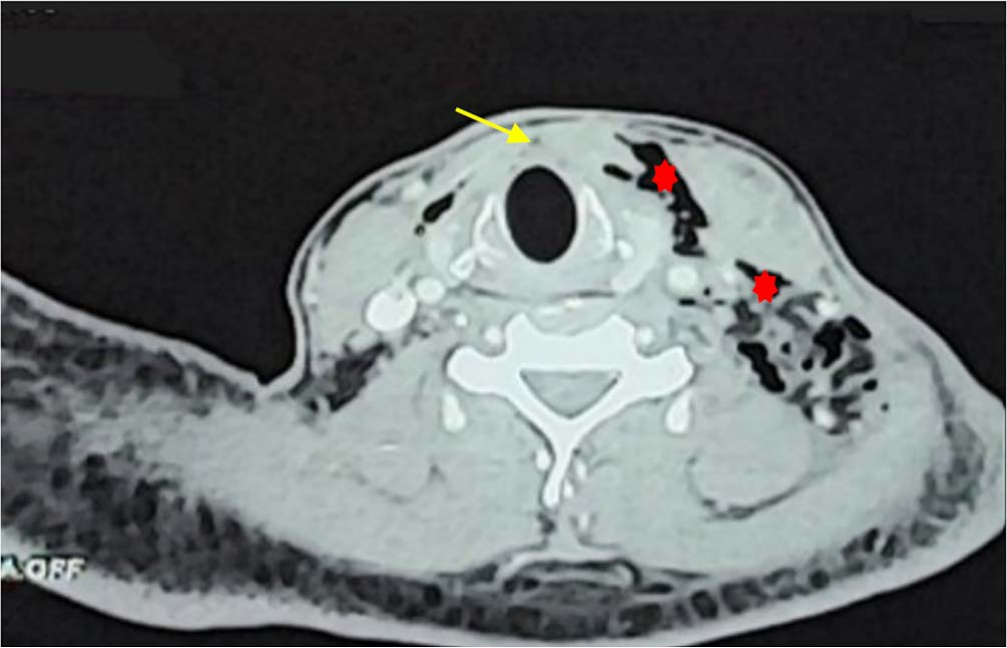
Axial contrast-enhanced cervical CT scans showing subhyoid fluid collections (yellow arrows) adjacent to the thyroid cartilage, associated with emphysema (red asterisks), consistent with cervicothoracic cellulitis.

**Figure 4:**
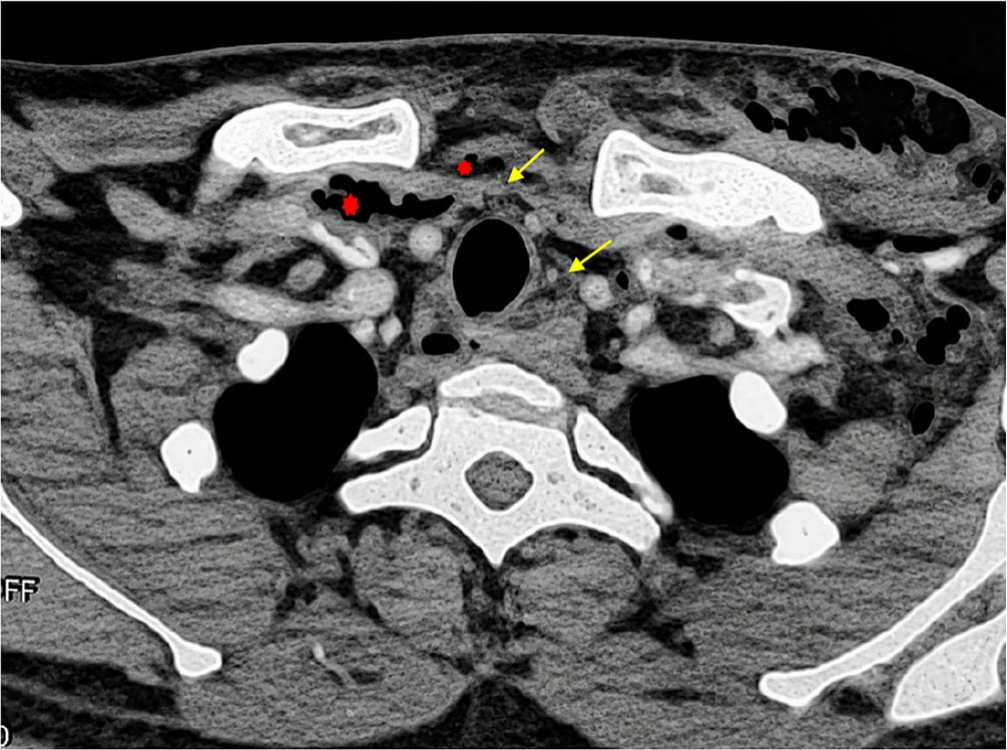
Axial contrast-enhanced thoracic CT images showing retrosternal mediastinal and left paratracheal collections (yellow arrows) associated with emphysematous infiltration of the mediastinal fat (red asterisks), consistent with descending necrotizing mediastinitis of cervical origin.

Contrast-enhanced cervicothoracic CT demonstrated extensive cervical cellulitis with multiple collections extending into the superior mediastinum, consistent with DNM, along with cervical emphysema and air tracking anterior to the thyroid cartilage (Figs 3 and 4).

**Figure 5:**
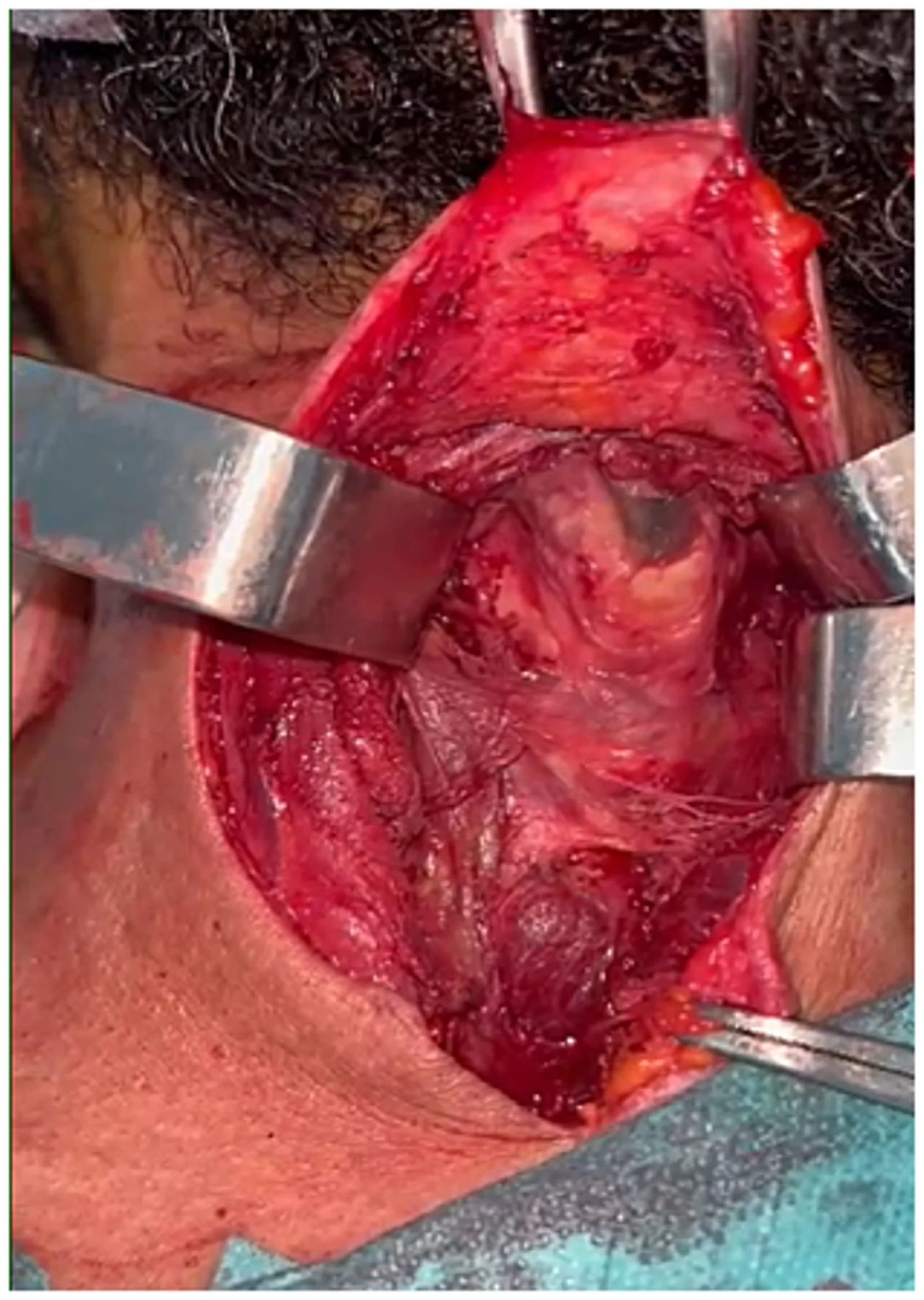
Per-operative image after drainage of cervicothoracic collections, showing a residual mild necrotic lesion of the thyroid cartilage*.*

Emergency surgery was performed jointly by otolaryngology and thoracic surgeons. Cervicotomy allowed drainage of multiple cervical abscesses, extensive debridement of necrotic tissue, and drainage of the superior mediastinum through the same approach, followed by saline irrigation. Delbet drains were placed for cervical drainage, and gauze packing was inserted at the suprasternal notch and behind the manubrium. The wound was partially closed. Tracheostomy secured the airway, and a nasogastric tube was inserted for enteral feeding (Fig. 5).

Cultures grew *Streptococcus pyogenes*, *Klebsiella pneumoniae*, and *Candida albicans*. Antimicrobial therapy consisted of imipenem, metronidazole, colistin, vancomycin, and fluconazole.

**Figure 6:**
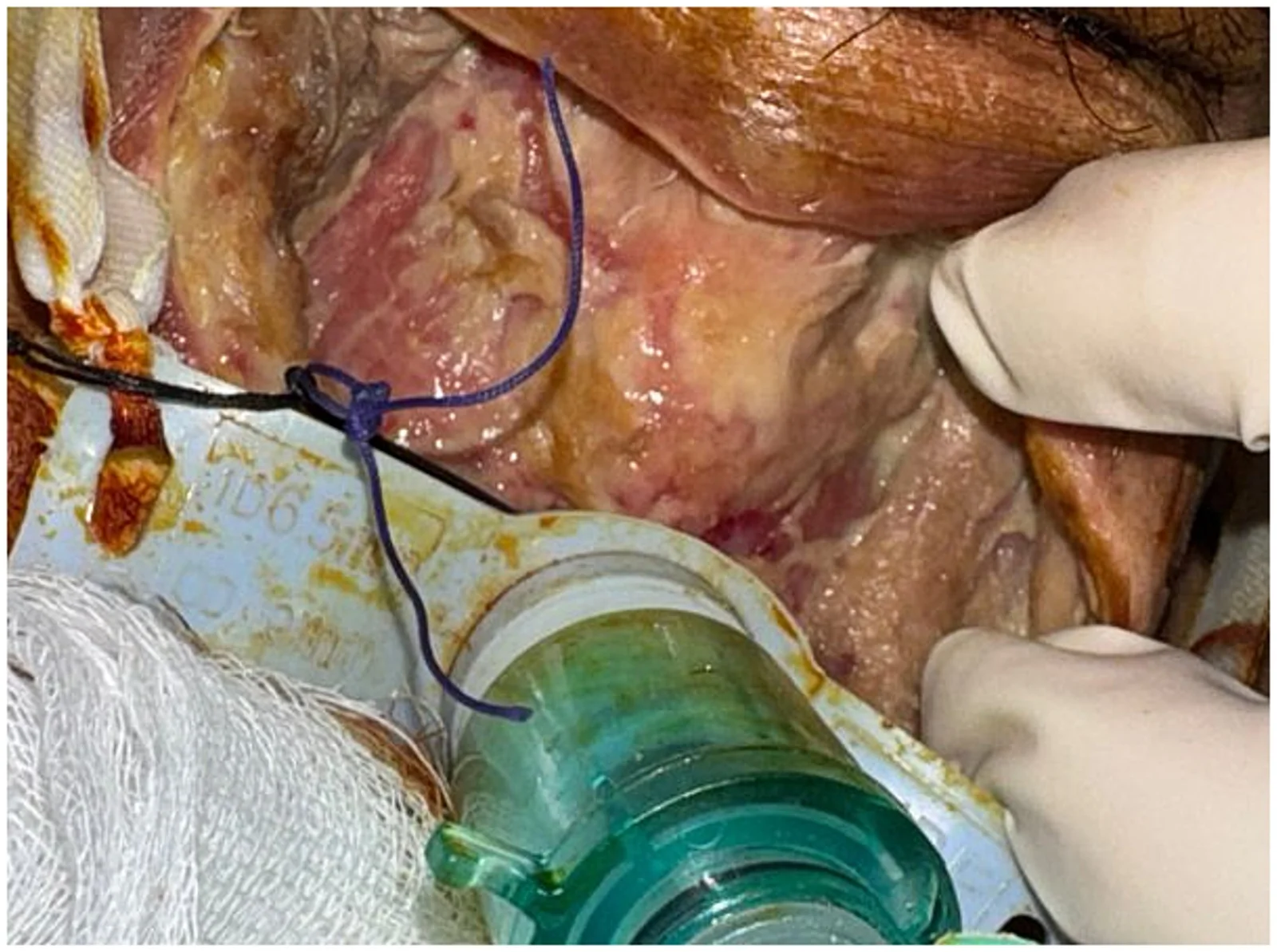
Day 4 postoperative image of the surgical site showing fibrin deposition with no evidence of pus, and the tracheostomy tube in place*.*

The postoperative course required prolonged intensive care and mechanical ventilation. No further debridement was necessary. Clinical and laboratory improvement was progressive. The patient was discharged from the intensive care unit after 20 days and from the otolaryngology department 15 days later. The nasogastric tube was maintained for 5 weeks to allow complete healing before oral feeding was resumed (Fig. 6).

## Discussion

DNM is an uncommon but highly severe complication of deep neck infections, with rapid progression and significant mortality despite modern management. Mortality remains high, ranging from 9% to 40%, which highlights the importance of early diagnosis and treatment [1–3].

Its pathophysiology is related to cervical fascial anatomy, particularly the retropharyngeal and ‘danger’ spaces, which allow infection to descend into the mediastinum under the influence of gravity and negative intrathoracic pressure [3, 6].

Although odontogenic infection is the most common source, pharyngeal fish bone impaction is an uncommon but recognised cause [4, 5]. In the present case, DNM developed despite apparently successful foreign body removal. The residual vallecular mucosal lesion seen on nasofibroscopy suggests that an occult mucosal injury allowed bacterial inoculation and delayed infection.

Poorly controlled diabetes is a recognized risk factor due to impaired immune response and altered microvascular function, contributing to rapid infection progression [2, 7]. The patient’s markedly elevated fasting glucose and HbA1c likely contributed to the severity and progression of disease.

Contrast-enhanced cervicothoracic CT is the imaging modality of choice because it accurately defines the extent of infection, identifies mediastinal involvement, and guides surgical planning [1, 8]. Our patient fulfilled the diagnostic criteria proposed by Estrera et al., including severe cervical infection, radiological evidence of mediastinal extension, intraoperative confirmation, and an identifiable cervical source [6]. In patients with cervical swelling, crepitus, fever, or chest symptoms after pharyngeal trauma, urgent CT should not be delayed.

Successful management depends on early multidisciplinary treatment combining broad-spectrum antimicrobial therapy, aggressive surgical drainage, airway protection, and intensive care support [4, 8, 9]. In this case, the infection was confined to the superior mediastinum and was adequately drained through a cervical approach, avoiding the need for thoracic drainage. Tracheostomy and enteral nutrition were essential to secure the airway and support healing. No reoperation was required, and the patient recovered progressively, illustrating the value of timely coordinated care [10].

Microbiological cultures revealed a polymicrobial infection with *S. pyogenes*, *K. pneumoniae*, and *C. albicans*. DNM is typically polymicrobial, reflecting the mixed flora of the upper aerodigestive tract [3]. Isolation of *C. albicans* in critically ill patients has been reported and may justify antifungal therapy in selected cases.

Beyond poorly controlled diabetes, risk factors for progression to DNM include delayed diagnosis, immunosuppression, and polymicrobial infections spreading along cervical fascial planes. Early recognition of red flags rapid neck swelling, crepitus, chest pain, or radiological mediastinal extension is essential for timely multidisciplinary care. A recent scoping review supports combined cervical-transthoracic drainage with early imaging and broad-spectrum antibiotics as the standard approach for optimal outcomes [9].

## Conclusion

Descending necrotizing mediastinitis secondary to a pharyngeal fish bone is a rare but life-threatening condition. This case highlights that delayed severe infections may occur even after apparent foreign body removal, particularly in patients with risk factors such as poorly controlled diabetes mellitus.

Early diagnosis based on clinical suspicion and contrast-enhanced CT imaging is essential. Prompt and aggressive management combining surgical drainage, intensive care support, and broad-spectrum antimicrobial therapy remains the cornerstone of treatment.

The presence of polymicrobial multidrug-resistant flora associated with *C. albicans* underscores the importance of microbiologically guided therapy, including antifungal treatment when indicated.

Timely multidisciplinary management is crucial to improving outcomes in this potentially fatal condition.
